# Distinguishing Japanese Spotted Fever and Scrub Typhus, Central Japan, 2004– 2015

**DOI:** 10.3201/eid2409.171436

**Published:** 2018-09

**Authors:** Eiichiro Sando, Motoi Suzuki, Shungo Katoh, Hiromi Fujita, Masakatsu Taira, Makito Yaegashi, Koya Ariyoshi

**Affiliations:** Nagasaki University, Nagasaki, Japan (E. Sando, M. Suzuki, S. Katoh, K. Ariyoshi);; Kameda Medical Center, Kamogawa, Japan (E. Sando, M. Yaegashi);; Mahara Institute of Medical Acarology, Anan, Japan (H. Fujita);; Chiba Prefectural Institute of Public Health, Chiba, Japan (M. Taira)

**Keywords:** Japanese spotted fever, scrub typhus, tsutsugamushi disease, *Orientia tsutsugamushi*, *Rickettsia japonica*, Rickettsia, Japan

## Abstract

Japanese spotted fever (JSF) and scrub typhus (ST) are endemic to Japan and share similar clinical features. To document the clinical and epidemiologic characteristics that distinguish these 2 rickettsial diseases, during 2004–2015 we recruited 31 JSF patients, 188 ST patients, and 97 nonrickettsial disease patients from the southern Boso Peninsula of Japan. JSF occurred during April–October and ST during November–December. Patients with JSF and ST were significantly older and more likely to reside in wooded areas than were patients with nonrickettsial diseases. Spatial analyses revealed that JSF and ST clusters rarely overlapped. Clinical findings more frequently observed in JSF than in ST patients were purpura, palmar/plantar rash, hyponatremia, organ damage, and delayed defervescence after treatment. Although their clinical features are similar, JSF and ST differ in seasonality, geographic distribution, physical signs, and severity. Because a considerable percentage of patients did not notice rash and eschar, many rickettsial diseases might be underdiagnosed in Japan.

Two rickettsial diseases are endemic to Japan, scrub typhus (ST) and Japanese spotted fever (JSF). ST, which is also called tsutsugamushi disease (*1*), was first reported in central Japan in 1878 (*2*). ST is caused by the miteborne pathogen *Orientia tsutsugamushi.* According to the national surveillance data of notifiable diseases in Japan, during 2004–2015, the number of reported ST cases was nearly constant; each year on average, 396 ST cases and 2 deaths (case-fatality rate 0.5%) were reported (*3*). ST was originally believed to be confined to the Asia–Pacific region; however, ST has recently been reported in Kenya (*4*) and southern Chile (*5*). In 1984, JSF was identified in western Japan (*6*). JSF is caused by the tickborne pathogen *Rickettsia japonica* (*7*). Except for a case reported in South Korea (*8*), JSF is endemic nearly exclusively to the central and western portions of Japan (*3*). Recently, the number of reported JSF cases in this region increased, from 66 in 2004 to 215 in 2015, and the case-fatality rate increased from 1.5% to 2.3% (*3*); thus, JSF is a public health concern. Although JSF and ST have been reported in several prefectures in Japan, the areas of endemicity rarely overlap at the district level (*3*). One of the rare districts to which both JSF and ST are endemic is the southern Boso Peninsula, Chiba Prefecture, in central Japan.

The typical signs and symptoms of JSF and ST are similar (e.g., fever, rash, and eschar), although in patients with ST, the frequency of rash varies from 14% to 93% and of eschar from 8% to 93% (*9**–**13*). For a few patients with JSF and ST, severe conditions develop (*14**–**17*). However, clinical information regarding JSF has been limited by lack of an appropriate case definition, lack of in-depth information, and studies involving small sample sizes (*14**,**16*). The clinical features observed in patients with JSF and ST are not comparable across studies because of the different enrollment criteria and nonstandardized case definitions. To clarify the clinical and epidemiologic characteristics of JSF and ST patients by using stringent laboratory confirmation methods and to identify the factors that distinguish the 2 diseases, we conducted a multicenter study in the southern Boso Peninsula in central Japan, an area of high JSF and ST endemicity. The study was approved by the institutional review boards of the Kameda Medical Center and the Awa Regional Medical Center.

## Methods

### Study Design and Setting

The southern Boso Peninsula is a predominantly rural mountainous region with a long coastline facing the Pacific Ocean and Tokyo Bay. According to the census, the total population in 2015 was 350,000 and 35.4% of the residents were >65 years of age. We conducted prospective and retrospective case series reviews at 3 medical facilities: Kameda Medical Center (865 acute beds), Awa Regional Medical Center (149 beds), and Kameda Family Clinic–Tateyama (no beds).

### Study Period and Entry Criteria

We prospectively enrolled patients from January 1, 2011, through December 31, 2015. We collected clinical, epidemiologic, and laboratory data from the patients who visited the study hospitals and exhibited signs and symptoms compatible with rickettsial disease. The patients were suspected to have rickettsial disease if they had any of the following clinical signs or symptoms without other apparent causes: fever, rash, eschar, respiratory symptoms, altered mental status, lymphadenopathy, neurologic abnormalities, systematic pain, chills/rigors, headache, or malaise. Using the same enrollment criteria, we also retrospectively collected data from patients who visited the study hospitals from January 1, 2004, through December 31, 2010, and who were not included in the prospective data collection. We used a standardized format to extract clinical and epidemiologic information from electronic medical records.

### Laboratory Methods

All blood samples were sent to a commercial laboratory (SRL, Inc., Tokyo) for an indirect immunofluorescence assay (IFA) to identify the *O. tsutsugamushi* serotypes Kato, Karp, and Gilliam; the antigens were provided by Denka Seiken Co., Ltd. If JSF was suspected, the samples were sent to the Chiba Prefectural Institute of Public Health for IFA to identify the *O. tsutsugamushi* serotypes Kato, Karp, Gilliam, Irie/Kawasaki, Hirano/Kuroki, and *R. japonica* (YH strain). The blood samples collected during 2009 and 2010 were also sent to the Ohara Research Laboratory (Fukushima City, Japan), and samples collected during 2014 were sent to the Mahara Institute of Medical Acarology (Anan, Japan) for an indirect immunoperoxidase assay to identify 6 *O. tsutsugamushi* serotypes (the previously mentioned 5 serotypes plus the serotype Shimokoshi), *R. japonica* (Aoki strain), and *R. typhi* (*18*). The type-specific whole rickettsial particles were used as antigens in the IFA and immunoperoxidase assays. Serum samples were diluted from 1:40 to 1:40,960 for immunoperoxidase assays and from 1:10 (or 20) to 1:10,240 for IFA. The titer was expressed as the reciprocal of the highest dilution. Nested PCR assays were performed to identify the 56-kDa antigen of *O. tsutsugamushi* and the 17-kDa genus-common antigen of *R. japonica* from eschars at the Chiba Prefectural Institute of Public Health (Chiba, Japan) or Kameda Medical Center (Kamogawa, Japan) (*19**,**20*).

### Case Definitions and Data Collection

A patient’s rickettsial status was defined as confirmed if the PCR result from the eschar was positive for any rickettsiae or if a >4-fold increase in the IgM or IgG titer of IFA or immunoperoxidase assay was observed in paired serum samples (i.e., acute and convalescent phases). A patient’s status that did not fulfill the criteria for confirmed was defined as probable if the IgM titer of IFA or immunoperoxidase assay was >80 for JSF or ST. A patient’s status was defined as possible if the clinical course was compatible with that of JSF or ST but the laboratory test results did not fulfill the criteria for either confirmed or probable. A patient was defined as having a nonrickettsial disease if a diagnosis of an infectious or noninfectious disease other than a rickettsial disease was confirmed. We excluded from analysis those patients who were classified as having possible cases or a diagnosis of murine typhus or concurrent JSF and ST infection.

Traditionally, in Japan, fever, rash, and eschar have been considered the triad of JSF and ST. We classified the triad into 3 categories: 1) “chief complaint” if any of the signs were the reason for the visit; 2) “upon history collection” if patients noticed the signs but had not complained until the physician asked; and 3) “physical exam” if the signs were objectively identified at the initial physical examination.

### Statistical Analyses

The clinical and epidemiologic characteristics of the patients were summarized and compared according to the 3 categories (i.e., JSF, ST, and nonrickettsial diseases). We used χ^2^ or Fisher exact tests to compare characteristics of the patients by disease category. We computed odds ratios (ORs) with 95% CIs by using logistic regression models.

The patients’ home addresses were geocoded and plotted on maps by using ArcGIS version 10.4.1 (Esri, Redlands, CA, USA). We calculated the population density and land use percentage within a radius of 500 m based on the census data and compared the 3 categories by using the Mann-Whitney U test. The Kulldorff scan statistics tool (SaTScan version 9.4.4) was used to identify the geographic clusters of JSF and ST (*21*). All tests were 2-tailed, and p<0.05 was considered statistically significant. All clinical data analyses were performed by using STATA version 13.0 (StataCorp LLC, College Station, Texas, USA).

## Results

### Laboratory Confirmation

**Figure 1:**
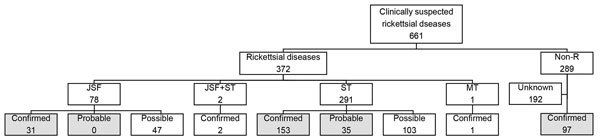
Numbers of patients with rickettsial or nonrickettsial diseases, Japan, 2004–2015. Of 43 patients tested by immunofluorescence and immunoperoxidase assays, 4 fulfilled the criteria for having confirmed JSF, 7 for confirmed ST, and 7 for probable ST. Gray shading indicates the cases included in the main analysis. JSF, Japanese spotted fever; MT, murine typhus; non-R, nonrickettsial disease; ST, scrub typhus.

A total of 661 patients were enrolled in the study: 303 by prospective and 358 by retrospective data collection. Overall, 42% of the patients were female, and the mean age was 60 years. Of the 50 patients whose eschars were tested by nested PCR, 8 were positive for *R. japonica* DNA and 29 were positive for *O. tsutsugamushi* DNA. The *O. tsutsugamushi* serotypes were identified in 22 patients; 16 were the Irie/Kawasaki type and 6 were the Hirano/Kuroki type. All patients were tested by an IFA at least 1 time, and paired blood samples were available for 304 (46%) patients. The median time from acute-phase sample collection to convalescent-phase sample collection was 14 days (interquartile range 11–17 days). Of the 304 patients, JSF was confirmed for 33 and ST was confirmed for 155. Of the 357 patients whose convalescent-phase samples were unavailable, none had probable JSF and 35 had probable ST. Three patients who did not fulfill the serologic criteria for having a rickettsial disease but whose eschar was positive for *O. tsutsugamushi* DNA were confirmed as having ST. Two patients fulfilled the criteria for having both JSF and ST, and 1 patient was confirmed to have murine typhus. Overall, our analysis included 31 patients with JSF, 188 patients with ST, and 97 patients with nonrickettsial diseases (Figure 1). The final diagnoses of the nonrickettsial diseases are shown in Technical Appendix Table 1).

### Seasonal and Geographic Distributions

**Figure 2:**
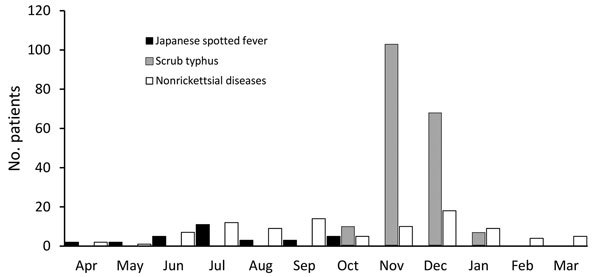
Number of patients with Japanese spotted fever, scrub typhus and nonrickettsial diseases, central Japan, by month, 2004–2015.

The seasonal distributions of JSF, ST, and nonrickettsial diseases are shown in Figure 2. All patients with JSF visited a medical facility during April–October; the numbers peaked slightly in July. Most (91%) patients with ST visited a medical facility in either November or December. No seasonal trend was observed for nonrickettsial diseases.

**Figure 3:**
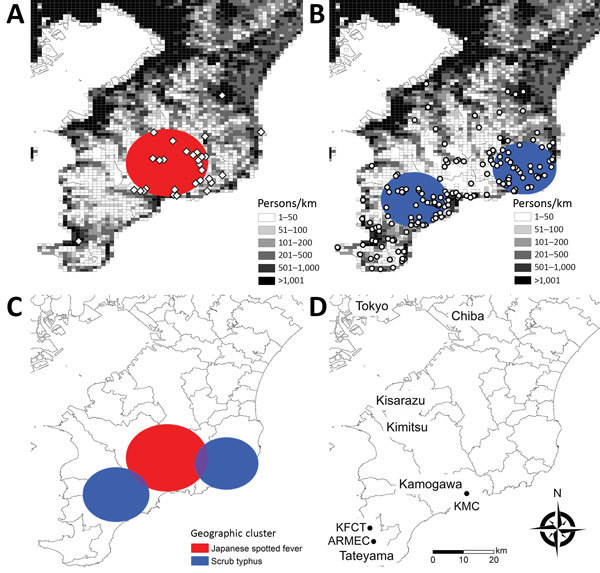
Geographic distribution and clusters of JSF and ST, Japan, 2004–2015. A) JSF; B) ST; C) geographic clusters of JSF and ST; D) locations of study facilities. White diamonds (JSF) and circles (ST) represent the locations of each patient’s address. Colored circles (black, JSF; gray, ST) represent statistically significant spatial clusters (p<0.05). The geographic distribution of the patients with nonrickettsial diseases, which were used for the cluster analysis as the reference, is shown in the Technical Appendix Figure). ARMEC, Awa Regional Medical Center; JSF, Japanese spotted fever; KFCT, Kameda Family Clinic Tateyama; KMC, Kameda Medical Center; ST, scrub typhus.

The geographic distributions are shown in Figure 3. We identified 1 JSF cluster (p<0.001) and 2 ST clusters (p = 0.013 and p = 0.041), and these clusters rarely overlapped. Patients with JSF and ST resided in less populated areas (population densities within a 500-m radius were 255/km^2^ and 295/km^2^, respectively) than patients with nonrickettsial diseases (904/km^2^; p<0.001). Patients with JSF and ST more frequently resided in wooded areas (proportions in forested area within a 500-m radius were 51% and 43%, respectively) than patients with nonrickettsial diseases (17%; p<0.001).

### Demographic and Clinical Features

The baseline characteristics of the patients are summarized in Table 1. The proportion of female patients did not differ among the 3 groups. Patients with JSF and ST were older than patients with nonrickettsial diseases; among patients in the oldest age group, JSF occurred more frequently than ST. Patients with JSF and ST were more frequently exposed to the natural outdoor environment than were patients with nonrickettsial diseases.

**Table 1:** Baseline characteristics of patients with JSF, ST, and nonrickettsial diseases, central Japan, 2004–2015*

Characteristic	JSF, no. (%), n = 31	ST, no. (%), n = 188	Non-R, no. (%), n = 97	JSF vs. non-R†			ST vs. non-R†			JSF vs. ST‡	
OR (95% CI)	p value	OR (95% CI)	p value	OR (95% CI)	p value
Female sex	16 (52)	85 (45)	35 (36)	1.9 (0.8–4.3)	0.127		1.5 (0.9–2.4)	0.14		1.3 (0.6–2.8)	0.509
Age, y, mean (SD)	73 (10)	65 (15)	57 (20)								
Age group, y											
<54	1 (3)	29 (15)	43 (44)	Reference			Reference			Reference	
55–64	7 (23)	48 (26)	14 (14)	21.5 (2.4–190.3)	0.006		5.1 (2.4–10.9)	<0.001		4.2 (0.5–36.1)	0.188
65–74	7 (23)	56 (30)	13 (13)	23.2 (2.6–205.9)	0.005		6.4 (3.0–13.7)	<0.001		3.6 (0.4–30.9)	0.239
>75	16 (52)	55 (29)	27 (28)	25.5 (3.2–203.3)	0.002		3.0 (1.6–5.8)	0.001		8.4 (1.1–66.8)	0.043
No exposure	1 (3)	18 (12)	32 (46)	Reference			Reference			Reference	
Living in/ stepped into mountainous areas	12 (40)	58 (38)	16 (23)	24.0 (2.9–201.2)	0.003		6.4 (2.9–14.3)	<0.001		3.7 (0.5–30.6)	0.162
Stepped into a bush	1 (3)	12 (8)	3 (4)	10.7 (0.5–217.2)	0.124		7.1 (1.8–28.6)	0.006		1.5 (0.1–26.4)	0.162
Farming	16 (53)	65 (42)	14 (20)	36.6 (4.4–303.4)	0.001		8.3 (3.6–18.7)	<0.001		4.4 (0.5–35.7)	0.162

*JSF, Japanese spotted fever; non-R, nonrickettsial diseases; OR, odds ratio; ST, scrub typhus.               †Non-R reference.              ‡ST reference.

Clinical characteristics of the patients are summarized in Table 2. The triad (i.e., fever, rash, and eschar) was commonly observed by physicians but not necessarily noticed by the patients. Fever was a primary sign; however, at the initial physical examination, body temperature was high in only 74% of patients with JSF and 73% with ST. Among patients who did not have a high body temperature at their initial physical examination, fever developed during hospitalization for 5 (71%) of 7 with JSF and 9 (38%) of 24 with ST. Although most patients had a rash, only 60% of patients with JSF and 44% with ST had noticed their rash. Moreover, only 45% of patients with JSF and 28% of patients with ST reported their rash. Most patients did not notice the presence of eschar.

**Table 2:** Clinical characteristics of patients with JSF, ST, and nonrickettsial diseases, central Japan, 2004–2015*

Characteristic	JSF, no. (%), n = 31	ST, no. (%), n = 188	Non-R, no. (%), n = 97								
				JSF vs. non-R†			ST vs. non-R†			JSF vs. ST‡	
				OR (95% CI)	p value		OR (95% CI)	p value		OR (95% CI)	p value
Chief complaint											
Fever	26 (84)	135 (72)	78 (80)	1.3 (0.4–3.7)	0.668		0.6 (0.3–1.1)	0.115		2.0 (0.7–5.6)	0.165
Rash	14 (45)	52 (28)	17 (18)	3.9 (1.6–9.3)	0.003		1.8 (1.0–3.3)	0.06		2.2 (1.0–4.7)	0.053
Eschar	0 (0)	5 (3)	1 (1)	Not applicable	1.000§		2.6 (0.3–22.8)	0.382		Not applicable	1.000§
Headache	1 (3)	29 (15)	5 (5)	0.6 (0.1–5.5)	0.661		3.4 (1.3–9.0)	0.016		0.2 (0–1.4)	0.101
Fatigue	3 (10)	35 (19)	6 (6)	1.6 (0.4–6.9)	0.511		3.5 (1.4–8.6)	0.007		0.5 (0.1–1.6)	0.233
At history collection											
Fever	27 (87)	148 (82)	81 (84)	1.3 (0.4–4.1)	0.712		0.8 (0.4–1.6)	0.586		1.5 (0.5–4.6)	0.473
Rash	18 (60)	74 (44)	24 (26)	4.3 (1.8–10.1)	0.001		2.2 (1.3–3.8)	0.099		2.0 (0.9–4.3)	0.664
Eschar	1 (4)	20 (12)	5 (6)	0.6 (0.1–5.5)	0.664		2.1 (0.8–5.9)	0.141		0.3 (0–2.2)	0.232
Headache	4 (25)	75 (56)	27 (59)	0.2 (0.1–0.8)	0.026		0.9 (0.5–1.8)	0.748		0.3 (0.1–0.9)	0.026
Fatigue	17 (94)	97 (84)	32 (94)	1.1 (0.1–12.6)	0.962		0.3 (0.1–1.4)	0.138		3.3 (0.4–26.5)	0.256
Physical examination findings											
BT >37.5°C	23 (74)	132 (73)	53 (59)	2.0 (0.8–5.0)	0.132		1.9 (1.1–3.2)	0.02		1.1 (0.4–2.5)	0.883
Hypotension¶	8 (26)	12 (6)	5 (5)	6.4 (1.9–21.4)	0.003		1.3 (0.4–3.7)	0.679		5.1 (1.9–13.8)	0.001
Heart rate >120 bpm	2 (7)	13 (8)	6 (7)	0.9 (0.2–4.9)	0.942		1.2 (0.4–3.2)	0.777		0.8 (0.2–3.8)	0.793
Respiratory rate >20/min	13 (54)	40 (39)	23 (45)	1.4 (0.5–3.8)	0.464		0.8 (0.4–1.5)	0.457		1.9 (0.8–4.6)	0.174
Altered mental status	5 (16)	14 (7)	15 (15)	1.1 (0.3–3.2)	0.929		0.4 (0.2–1.0)	0.038		2.4 (0.8–7.2)	0.121
Rash	30 (100)	181 (96)	52 (57)	Not applicable	<0.001§		19.4 (8.2–45.9)	<0.001		Not applicable	0.597§
Localized	0	3 (2)	6 (12)	Not applicable	0.079§		0.1 (0–0.5)	0.004		Not applicable	1.000§
Purpura	11 (44)	4 (2)	7 (8)	8.9 (2.9– 26.8)	<0.001		0.2 (0.1–0.9)	0.028		36.1 (10.1–128.3)	<0.001
Palms/soles	21 (84)	13 (7)	4 (5)	101.1 (23.3– 438.4)	0.001		1.4 (0.5–4.6)	0.537		70.3 (21.0–235.3)	<0.001
Eschar	24 (89)	163 (87)	18 (22)	28.0 (7.6– 103.7)	<0.001		23.8 (12.1–46.8)	<0.001		1.2 (0.3–4.2)	0.801
Lung involvment#	8 (26)	21 (11)	9 (9)	3.4 (1.2–9.8)	0.023		1.2 (0.5–2.8)	0.622		2.8 (1.1–7.0)	0.031

*BT, body temperature; involve, involvement; JSF, Japanese spotted fever; non-R, nonrickettsial diseases; OR, odds ratio; ST, scrub typhus.              †Non-R = reference.              ‡ST = reference.              §Fisher exact tests.              ¶Systolic blood pressure <90 mm Hg or vasopressor usage.              #Lung rales with pulmonary infiltrative shadow.

During physical examination, patients with JSF had hypotension more frequently than patients with ST (OR 5.1, 95% CI 1.9–13.8), but no significant difference was observed in the frequency of tachycardia and tachypnea. Considerably higher proportions of patients with JSF and ST than with nonrickettsial diseases had a rash and eschar; the mean ± SD size of the eschar was smaller in patients with JSF (5.8 ± 2.1 mm) than in patients with ST (9.7 ± 5.6 mm; p = 0.024). Purpura, palmar/plantar rash, and lung involvement were more frequently observed in patients with JSF than in those with ST. Prevalence of lymphadenopathy did not differ among the groups.

Patients with JSF and ST were less likely than patients with nonrickettsial diseases to have leukocytosis and anemia but more likely to have elevated aspartate aminotransferase and lactate dehydrogenase levels, hyponatremia, and urine occult blood (Table 3). Patients with JSF were more likely than patients with ST to have low platelet counts; elevated bilirubin, creatinine kinase, blood urea nitrogen, and creatinine levels; hyponatremia; and high C-reactive protein.

**Table 3:** Laboratory and treatment data for patients with JSF, ST, and nonrickettsial diseases central Japan, 2004–2015*

Characteristic	JSF, no. (%), n = 31	ST, no. (%), n = 188	Non-R, no. (%), n = 97								
				JSF vs. non-R†			ST vs. non-R†			JSF vs. ST‡	
				OR (95% CI)	p value		OR (95% CI)	p value		OR (95% CI)	p value
Laboratory data											
Leukocytes >9,800/μL	5 (16)	23 (12)	42 (45)	0.2 (0.1–0.7)	0.006		0.2 (0.1–0.3)	<0.001		1.4 (0.5–3.9)	0.556
Hb <11 g/dL (F) or <13.5 g/dL (M)	5 (16)	29 (16)	46 (49)	0.2 (0.1–0.6)	0.002		0.2 (0.1–0.3)	<0.001		1.0 (0.4–2.9)	0.939
Platelets <130,000/μL	22 (71)	59 (32)	28 (30)	5.7 (2.3–13.9)	<0.001		1.1 (0.6–1.8)	0.806		5.3 (2.3–12.2)	<0.001
Albumin <3.4 g/dL	14 (61)	37 (29)	31 (55)	1.3 (0.5–3.4)	0.653		0.3 (0.2–0.6)	0.001		3.8 (1.5–9.6)	0.004
AST >33 IU/L	29 (94)	154 (83)	46 (50)	14.5 (3.3–64.3)	<0.001		4.8 (2.8–8.4)	<0.001		3.0 (0.7–13.3)	0.145
ALT >42 IU/L	16 (52)	100 (54)	42 (46)	1.3 (0.6–2.9)	0.566		1.4 (0.8–2.3)	0.204		0.9 (0.4–2.0)	0.824
LDH >229 IU/L	30 (97)	179 (97)	70 (78)	8.6 (1.1–66.8)	0.040		8.5 (3.3–22.1)	<0.001		1.0 (0.1–8.7)	0.996
Total bilirubin >1.0 mg/dL	9 (29)	13 (7)	15 (17)	2.0 (0.8–5.1)	0.166		0.4 (0.2–0.8)	0.016		5.2 (2.0–13.6)	0.001
Direct bilirubin >0.4 mg/dL	4 (22)	7 (5)	16 (28)	0.8 (0.2–2.6)	0.652		0.1 (0.1–0.4)	<0.001		5.2 (1.3–19.9)	0.017
Creatine kinase >150 IU/L	19 (66)	46 (29)	21 (28)	4.9 (2.0–12.2)	0.001		1.2 (0.6–1.9)	0.861		4.6 (2.0–10.7)	<0.001
BUN >22 mg/dL	15 (48)	35 (19)	20 (22)	3.4 (1.4–8.0)	0.006		0.8 (0.5–1.6)	0.58		4.0 (1.8–8.9)	0.001
Creatinine >1.2 mg/dL	11 (35)	22 (12)	7 (8)	6.7 (2.3–19.4)	<0.001		1.6 (0.7–4.0)	0.277		4.1 (1.7–9.6)	0.001
Sodium <135 mEq/L	24 (77)	71 (39)	16 (17)	16.3 (6.0–44.3)	<0.001		3.0 (1.6–5.6)	<0.001		5.4 (2.2–13.2)	<0.001
Chloride <98 mEq/L	17 (55)	37 (22)	15 (16)	6.2 (2.5–15.3)	<0.001		1.4 (0.7–2.7)	0.302		4.4 (2.0–9.7)	<0.001
CRP >10 mg/dL	16 (52)	32 (18)	34 (40)	1.6 (0.7–3.7)	0.266		0.3 (0.2–0.6)	<0.001		5.0 (2.2–11.1)	<0.001
Urine protein	27 (87)	116 (75)	34 (62)	4.2 (1.3–13.6)	0.018		1.8 (1.0–3.5)	0.068		2.3 (0.7–6.9)	0.148
Urine blood	29 (94)	122 (79)	31 (56)	11.2 (2.4–51.8)	0.002		2.9 (1.5–5.5)	0.002		3.9 (0.9–17.3)	0.071
Treatment and prognosis											
Duration of illness§ <5 d	16 (59)	74 (39)	24 (27)	4.0 (1.6–9.8)	0.002		1.8 (1.0–3.1)	0.039		2.2 (1.0–5.1)	0.054
Treatment: MINO/DOXY	31 (100)	180 (99)	42 (91)	Not applicable	0.144¶		17.1 (1.9–157.4)	0.012		Not applicable	1.000¶
Delayed defervescence#	11 (37)	17 (13)	30 (67)	0.3 (0.1–0.8)	0.012		0.1 (0–0.2)	<0.001		3.8 (1.5–9.3)	0.004
Hospitalization	28 (90)	104 (55)	80 (82)	2.0 (0.5–7.3)	0.302		0.3 (0.1–0.5)	<0.001		7.5 (2.2–25.7)	0.001
30-d mortality	0	1 (1)	2 (2)	Not applicable	1.000§		0.3 (0–3.0)	0.287		Not applicable	1.000¶

*ALT, alanine aminotransferase; AST, aspartate aminotransferase; BT, body temperature; BUN, blood urea nitrogen; CRP, C-reactive protein; DOXY, doxycycline; F, female patients; Hb, hemoglobin; JSF, Japanese spotted fever; LDH, lactate dehydrogenase; M, male patients; MINO, minocycline; non-R, nonrickettsial diseases; OR, odds ratio; ST, scrub typhus.              †Non-R = reference.              ‡ST = reference.              §Duration from the onset of symptoms to the first diagnostic test.              ¶Fisher exact tests.              #>3 d to decline of fever <37.3°C.

Patients with JSF required hospitalization more frequently than did patients with ST; these associations did not change after we adjusted for age. Patients with JSF tended to visit a medical facility earlier than did patients in the other groups. JSF and ST were successfully treated in patients who received tetracycline; a 51-year-old patient receiving psychiatric care died of ST, but no patient died of JSF. The time to defervescence after treatment was longer for patients with JSF than for patients with ST.

## Discussion

By using standardized laboratory definitions for diagnosis, we determined that the clinical and epidemiologic characteristics of JSF and ST in Japan differed by seasonality, geographic distribution, physical signs, and severity. JSF and ST showed distinct seasonal patterns. JSF occurred during April–October and peaked slightly in July, whereas most ST occurred during November–December. JSF and ST were distributed in less populated and more wooded areas, although their geographic clusters rarely overlapped. Patients with JSF were more likely than patients with ST to have purpura, palmar/plantar rash, and organ damage and to be hospitalized.

The different seasonal distribution of JSF and ST observed in our study can be explained by the ecology of the vectors (*3**,**22*) as follows: 1) *Haemaphysalis flava* and *H. longicornis* ticks, which transmit *R. japonica,* are active from spring until autumn in Chiba (*23*); 2) *Leptotorombidium scutellare* mites, which transmit the Irie/Kawasaki (and Hirano/Kuroki) serotypes of *O. tsutsugamushi,* are active in autumn and early winter (*24*) and unable to survive the winter; and 3) *L. pallidum* mites, which transmit the Karp and Gilliam serotypes of *O. tsutsugamushi,* are active from October through May (*24*). The difference in geographic distributions of JSF and ST may also be explained by the different distribution of the reservoirs in our study settings. Sika deer are wild hosts of ticks, and their distribution overlaps with that of ticks (*25*). The cluster of JSF identified in our study overlapped with the distribution of sika deer and Reeves’s muntjacs, which are related to sika deer (*26**,**27*). In contrast, the field rat, which is the primary host of the *Leptotrombidium* mite, is spreading throughout this area, which may explain the wide distribution of ST. Although our data are limited, similar patterns (i.e., the clustering of JSF and relatively wide distribution of ST) were also observed in other prefectures (*28**,**29*). Further studies are needed to establish the temporal and geographic associations among the vectors, reservoirs, and rickettsial pathogens.

Although the clinical features of patients in this study with JSF and ST were similar, some clinical findings were characteristic of JSF. Patients with JSF more frequently had rashes on the palms/soles, purpura, and small eschars. Moreover, the following severe conditions occurred more frequently among patients with JSF than among those with ST: hypotension, low platelet counts, and increased creatinine levels. Rickettsiae invade and proliferate within vascular endothelial cells and cause a vasculitis-like systemic disease (*30*). In a study by Tai et al., cytokine and chemokine levels were higher in patients with JSF than in patients with ST, but no significant association was observed between cytokine levels and the clinical severity of disease (*17*). Although previous human and animal model studies have revealed the pathogenic mechanisms of severe rickettsial infections (*31**,**32*), the mechanisms of severe JSF remain not fully understood. Of note, the clinical severity of ST may differ according to the *Orientia* serotype. According to a systematic review, the mortality rate from ST substantially varied according to patients’ age, co-occurring conditions, and regional *Rickettsia* strains (*33*). Our findings of ST in regions where Irie/Kawasaki type and Hirano/Kuroki type are endemic may not be directly applicable to other settings in which other serotypes are endemic, such as Akita and Niigata in northern Japan.

Fever has been considered one of the typical signs of JSF and ST. Most patients in our study had a high body temperature during the clinical course of their illness; however, fever was not apparent at the time of initial physical examination for 26% of patients with JSF and 27% of patients with ST. Although rash and eschar were commonly observed by the physicians, more than half of the patients did not notice these signs. Consequently, 33% of the patients with JSF and 34% of the patients with ST received incorrect diagnoses during their first medical visit. Furthermore, fewer clinicians were aware of JSF than of ST (*34*). These findings indicate that a substantial number of rickettsial diseases may be underdiagnosed in Japan.

Because of the difficulties associated with locating patients to collect blood samples during the convalescent phase of illness, previous studies have relied on laboratory confirmation that uses acute-phase samples with variable cutoff IgM titers without considering local endemicity, which may have resulted in misclassification (*35*). In this study, we used the IFA or immunoperoxidase IgM titer of >80 as a cutoff for the diagnosis of JSF and ST for patients for whom convalescent-phase samples were unavailable. To determine the optimum cutoff titer in our setting, we collected blood samples from patients with nonrickettsial diseases and confirmed that the highest IgM titer for *R. japonica* was <20 and that for *O. tsutsugamushi* was 10 (Technical Appendix Table 2). Therefore, our diagnostic criteria must be very specific.

During the acute phase of the disease, sensitivity of the IFA is quite low; in our study, an elevated IgM titer by IFA was observed in the acute-phase samples of only 2 (6.5%) of 31 patients with JSF and 73 (38.8%) of 188 patients with ST. Hence, physicians may overlook these diseases if their diagnosis relies on IgM titer by IFA during the early phase. Furthermore, the ST serotypes affect the sensitivity of the IFA. In our study, of the 22 patients for whom serotype was identified, 16 serotypes were Irie/Kawasaki and 6 serotypes were Hirano/Kuroki. In Japan, health insurance covers IFAs for the standard serotypes Kato, Karp, and Gilliam only but not for serotypes Irie/Kawasaki and Hirano/Kuroki, which may not be cross-reactive to the standard serotypes (*22*). In our study population, use of IFAs to test for the standard serotypes could have led to underdiagnosis of ST for ≈5% of the patients because 2 patients with the Irie/Kawasaki and Hirano/Kuroki serotypes did not react to any of the standard serotypes.

Our study has limitations because we did not include the other 2 hospitals in the southern Boso Peninsula. However, our study sites are the only medical facilities in the district that have infectious disease specialists. Most patients with acute disease and fever in this district are expected to visit our clinic and hospitals. Thus, we believe that the effect of selection bias was minimal. Because our study is a combined prospective and retrospective case-series, the quality of the information may have differed between the prospectively and the retrospectively identified patients. However, we used an identical case definition throughout the study, and further analyses indicated that the clinical and epidemiologic characteristics did not differ between 2 groups (Technical Appendix Table 3).

In conclusion, although JSF and ST share similar clinical features, in Japan the 2 diseases differ in seasonality, geographic distribution, physical signs, and severity. Patients with rickettsial diseases often do not notice their rash and eschar, and the sensitivity of the serologic test can be low during the acute phase of illness. A substantial number of rickettsial diseases may be underdiagnosed.

Technical Appendix Number of and distribution of cases of nonrickettsial disease, central Japan, 2004–2015.
