# Supplementary material for: Distinguishing Japanese Spotted Fever and Scrub Typhus, Central Japan, 2004– 2015
Source: Emerg Infect Dis. 2018 Sep;24(9):1633–41. doi: 10.3201/eid2409.171436 (PMC6106405; doi:10.3201/eid2409.171436)
Supplement: Technical Appendix — Number of and distribution of cases of nonrickettsial disease, central Japan, 2004–2015. [file 17-1436-Techapp-s1.pdf]

Article DOI: https://doi.org/10.3201/eid2409.171436

Distinguishing Japanese Spotted Fever from Scrub Typhus, Central Japan, 2004–2015

Technical Appendix

Technical Appendix Table 1. Diagnosis of nonrickettsial diseases*

Diagnosis	N
Bacterial	
Bacteremia†	16
Cellulitis	5
Pneumonia‡	5
Cat scratch disease	4
Acute cholangitis	1
Acute prostatitis	1
Hemolytic streptococcus infection	1
Meningitis	1
Miliary tuberculosis	1
Mycoplasma	1
Viral	
EB, CMV infection	10
Influenza	3
Adenovirus infection	1
Varicella	1
Measles	1
Hepatitis A infection	1
Herpes zoster	1
Acute HIV infection	1
Neoplasm	
Multiple myeloma	2
Malignant lymphoma	2
Lung cancer	1
Collagen Vascular Diseases	
Adult-onset Still's disease	10
Systemic lupus erythematosus	4
ANCA-related vasculitis	3
Dermatomyositis/Polymyositis	3
Giant cell arthritis	2
Polymyalgia rheumatica	2
Sjögren's related meningitis	1
RS3PE syndrome	1
Behçet's disease	1
Eosinophilic pneumonia	1
Others	
Erythema nodosum	4
Stevens-Johnson syndrome	1
Subacute thyroiditis	1
Hypersensitivity pneumonitis	1
Hypothyroidism	1
Erythema multiforme	1

*EBV, Epstein-Barr virus; CMV, cytomegalovirus; HIV, human immunodeficiency virus; ANCA, anti-neutrophil cytoplasmic antibody; RS3PE, remitting seronegative symmetric synovitis with pitting edema.
†Infectious endocarditis cases are included.
‡Only laboratory-confirmed cases are included.

Technical Appendix Table 2. Antibody titers for *Rickettsia japonica* and *Orientia tsutsugamushi* in the patients with nonrickettsial diseases.

	Antibody titer*										
Serotype	<10	10	20	40	80	160	320	640	1280	2560	5120
Acute phase IgM											
R. japonica (n = 26)	26	-	0	0	0	0	0	0	0	0	0
O. tsutsugamushi (n = 93)											
Kato	91	2	0	0	0	0	0	0	0	0	0
Karp	90	3	0	0	0	0	0	0	0	0	0
Gilliam	90	3	0	0	0	0	0	0	0	0	0
Acute phase IgG											
R. japonica (n = 26)	25	-	1	0	0	0	0	0	0	0	0
O. tsutsugamushi (n = 93)											
Kato	87	3	1	2	0	0	0	0	0	0	0
Karp	85	4	3	1	0	0	0	0	0	0	0
Gilliam	87	0	3	2	1	0	0	0	0	0	0

*The titer was expressed as the reciprocal of the highest dilution.

Technical Appendix Table 3. Characteristics of the patients by the enrollment status.

	Japanese spotted fever			Scrub typhus		
Characteristic	Retrospective (n = 11)	Prospective (n = 20)	p-value†	Retrospective (n = 90)	Prospective (n = 98)	p-value†
Female gender	7 (64)	9 (45)	0.458	38 (42)	47 (48)	0.465
Age, y						
≤54	0 (0)	1 (5)	0.798	15 (17)	14 (14)	0.665
55 – 64	2 (18)	5 (25)		25 (28)	23 (23)	
65 – 74	2 (18)	5 (25)		23 (26)	33 (34)	
≥75	7 (64)	9 (45)		27 (30)	28 (29)	
Exposure to the natural outdoor environment						
No exposure	0 (0)	1 (5)	0.753	7 (11)	11 (13)	0.294
Living in/stepped into mountainous areas	3 (30)	9 (45)		20 (30)	38 (43)	
Stepped into a bush	0 (0)	1 (5)		7 (11)	5 (6)	
Farming	7 (70)	9 (45)		32 (48)	33 (38)	
Chief complaint						
Fever	9 (82)	17 (85)	1.000	61 (68)	74 (76)	0.259
Rash	4 (36)	10 (50)	0.707	26 (29)	26 (27)	0.746
Eschar	0 (0)	0 (0)	NA	2 (2)	3 (3)	1.000
Headache	1 (9)	0 (0)	0.355	10 (11)	19 (19)	0.157
Fatigue	1 (9)	2 (10)	1.000	9 (10)	26 (27)	0.005
Upon history collection						
Fever	10 (91)	17 (85)	1.000	69 (81)	79 (82)	0.85
Rash	6 (60)	12 (60)	1.000	36 (46)	38 (41)	0.539
Eschar	1 (11)	0 (0)	0.333	7 (9)	13 (14)	0.344
Headache	1 (17)	3 (30)	1.000	28 (52)	47 (59)	0.48
Fatigue	7 (88)	10 (100)	0.444	35 (76)	62 (89)	0.122
Physical exams						
BT >37.5°C	9 (82)	14 (70)	0.676	65 (77)	67 (69)	0.242
Hypotension‡	1 (9)	7 (35)	0.203	1 (1)	11 (11)	0.005
Heart rate >120/bpm	1 (9)	1 (5)	1.000	8 (11)	5 (6)	0.25
Respiratory rate>20/min	7 (78)	6 (40)	0.105	11 (31)	29 (43)	0.294
Altered mental status	0 (0)	5 (25)	0.133	6 (7)	8 (8)	0.785
Rash	10 (100)	20 (100)	NA	85 (94)	96 (98)	0.262
Rash: localized	0 (0)	0 (0)	NA	2 (2)	1 (1)	0.607
Rash-purpura	6 (86)	5 (28)	0.021	2 (2)	2 (2)	1.000
Rash-palms/soles	5 (71)	16 (89)	0.548	3 (3)	10 (10)	0.086
Eschar	8 (89)	16 (89)	1.000	77 (86)	86 (89)	0.662
Lung involvement§	4 (36)	4 (20)	0.405	12 (13)	9 (9)	0.488
Laboratory data						
leukocyte >9800/μL	2 (18)	3 (15)	1.000	14 (16)	9 (9)	0.189
Hb <11 g/dL (F) or <13.5 g/dL(M)	1 (9)	4 (20)	0.631	15 (17)	14 (14)	0.687
Platelet <130,000/μL	9 (82)	13 (65)	0.429	22 (25)	37 (38)	0.06
Albumin <3.4 g/dL	2 (50)	12 (63)	1.000	17 (41)	20 (23)	0.038
AST >33 IU/L	10 (91)	19 (95)	1.000	72 (82)	82 (84)	0.846
ALT >42 IU/L	5 (45)	11 (55)	0.716	49 (56)	51 (52)	0.66

Characteristic	Japanese spotted fever Retrospective (n = 11)	Prospective (n = 20)	p-value†	Scrub typhus Retrospective (n = 90)	Prospective (n = 98)	p-value†
LDH >229 IU/L	11 (100)	19 (95)	1.000	89 (100)	90 (94)	0.029
Total bilirubin >1.0 mg/dL	1 (9)	8 (40)	0.106	6 (7)	7 (8)	1.000
Direct bilirubin >0.4 mg/dL	0 (0)	4 (40)	0.092	1 (2)	6 (8)	0.13
Creatine kinase >150 IU/L	4 (40)	15 (79)	0.051	14 (20)	32 (36)	0.035
BUN >22 mg/dL	3 (27)	12 (60)	0.135	12 (14)	23 (23)	0.132
Creatinine >1.2 mg/dL	2 (18)	9 (45)	0.241	8 (9)	14 (14)	0.365
Sodium <135 mEq/L	9 (82)	15 (75)	1.000	34 (40)	37 (38)	0.763
Chloride <98 mEq/L	5 (45)	12 (60)	0.477	16 (21)	21 (22)	1.000
C-reactive protein >10 mg/dL	6 (55)	10 (50)	1.000	18 (20)	14 (15)	0.338
Urine protein	8 (73)	19 (95)	0.115	47 (73)	69 (76)	0.851
Urine blood	10 (91)	19 (95)	1.000	53 (83)	69 (76)	0.325
Treatment and prognosis						
Duration of illness¶ <5 d	4 (50)	12 (63)	0.675	29 (32)	45 (46)	0.073
Treatment: MINO / DOXY	11 (100)	20 (100)	NA	86 (99)	94 (100)	0.481
Delayed defervescence#	3 (27)	8 (42)	0.466	12 (18)	5 (8)	0.12
Hospitalization	9 (82)	19 (95)	0.281	43 (48)	61 (62)	0.056
30-d mortality	0 (0)	0 (0)	NA	1 (1)	0 (0)	0.482

* NA, not applicable; BT, body temperature; leukocyte, leukocytes, Hb, hemoglobin; AST, aspartate aminotransferase; ALT, alanine aminotransferase; LDH, lactate dehydrogenase; BUN, blood urea nitrogen; MINO, minocycline; DOXY, doxycycline.
† Fisher exact tests
‡ Systolic blood pressure <90 mm Hg or vasopressor usage.
§ Lung rales with pulmonary infiltrative shadow.
¶ Duration from the onset of symptoms to the first diagnostic test.
# >3 d to decline of fever <37.3°C.

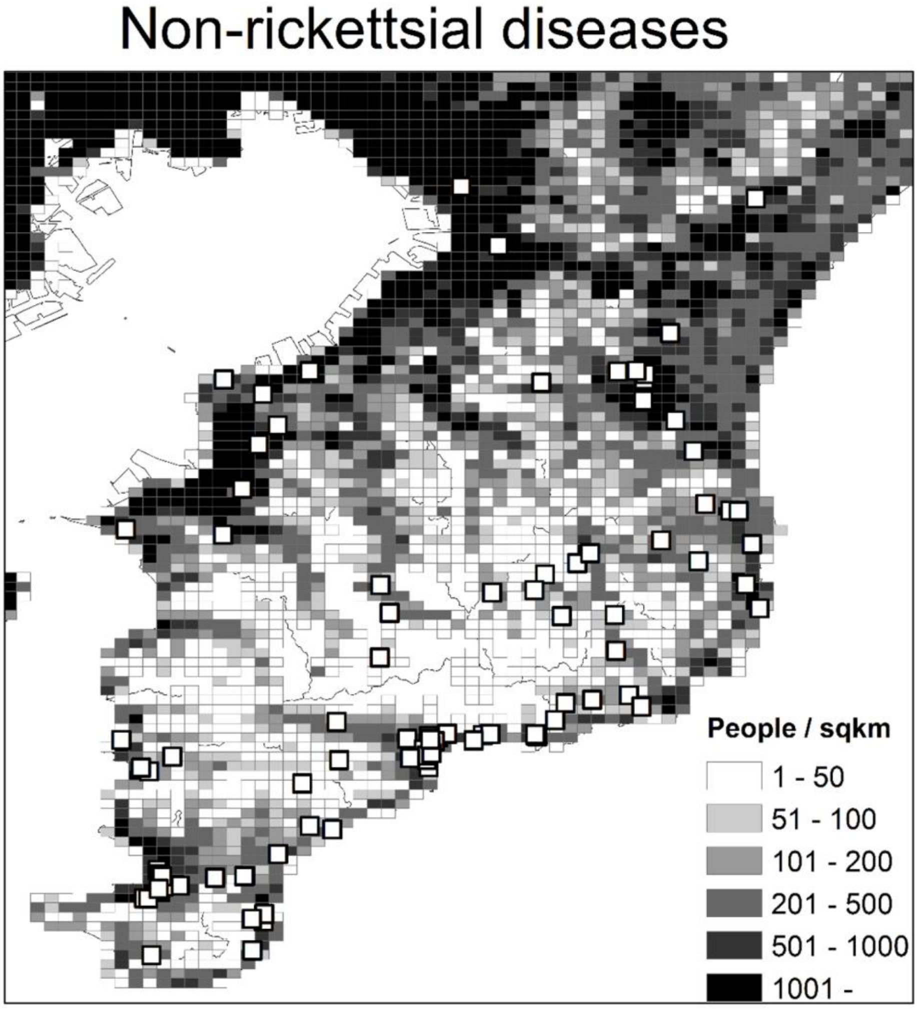

Technical Appendix Figure. Geographic distribution of nonrickettsial diseases. White boxes (nonrickettsial diseases) represent the locations of each patient’s address.
